# Supplementary material for: Developing a new cleavable crosslinker reagent for in-cell crosslinking
Source: Commun Chem. 2025 Jun 23;8:191. doi: 10.1038/s42004-025-01568-1 (PMC12185727; doi:10.1038/s42004-025-01568-1)
Supplement: Supplementary file 2 — Supplementary Information [file 42004_2025_1568_MOESM2_ESM.pdf]

Developing a new cleavable crosslinker reagent for in-cell crosslinking

Supplement

Organic Synthesis 2
General Information 2
Synthesis of DiSPASO 3
General Procedure 1: Synthesis of Aryl Bromide (GP-1)[2,3] 4
General Procedure 2: Reduction of Esters (GP-2) 5
General Procedure 3: Halogenation of Alcohols (GP-3) 6
General Procedure 4: Synthesis of Key Intermediate S5 (GP-4) 7
General Procedure 5: Sonogashira Cross-Coupling (GP-5) 8
General Procedure 6: Global Deprotection (GP-6) 9
General Procedure 7: Synthesis of NHS Protected Precursor S8 (GP-7) 10
General Procedure 8: Synthesis of NHP Protected Precursor (GP-8) 11
General Procedure 9: Synthesis of DiSPASO and DiPPASO (GP-9) 12
Supplemental figures 14
Supplemental tables 18
References 22

List of Figures

- ➔ Scheme 1: DiSPASO synthetic route.
- ➔ Scheme 2: DiPPASO synthetic route from common precursor S7.
- ➔ Figure S1: Single peptide evaluation of DiSPASO.
- ➔ Figure S2: Evaluation of picolyl concentration for click reaction and enrichment sensitivity in HeLa background.
- ➔ Figure S3: Optimization of Azide-S-S-biotin, sodium ascorbate and bead amount to achieve optimal click and enrichment performance.
- ➔ Figure S4: Comparison of different bead types.
- ➔ Figure S5: Confocal microscopy pictures of crosslinked HEK 293 cells using DiSPASO.
- ➔ Figure S6: Exemplary workflow of an MS/MS2 search with MS Annika 2.0 in Proteome Discoverer.
- ➔ Figure S7: Application of ASSB-DiSPASO enrichment strategies of spike-in ribosome samples.

List of Tables

- ➔ Table S1. Special reagents used for DiSPASO click-reaction, enrichment, and microscopy.

➔ Table S2: Fragment names, substitution, and monoisotopic masses of DiSPASO fragments used for crosslinking search.
➔ Table S3: Search parameters for linear and crosslink search. Parameters not listed here were left at default settings
➔ Table S4: IUPAC and supplier names of chemical compounds and their abbreviations used in this manuscript.

Organic Synthesis

General Information

General Procedures. All reactions were performed in round-bottom flasks or vials fitted with rubber septa and with magnetic stirring, unless otherwise stated. Reaction vessels were flushed with argon prior to use, unless otherwise stated. Liquids and solutions were transferred via syringe. All reactions were performed using anhydrous solvents obtained from Acros Organics, TCI or Sigma-Aldrich. Reaction progress was monitored by thin layer chromatography (TLC) performed on aluminum plates coated with silica gel F_{254} with 0.2 mm thickness. Chromatograms were visualized by fluorescence quenching with UV light at 254 nm or by staining using potassium permanganate, followed by heating. Flash column chromatography was performed using silica gel 60 (230-400 mesh, Merck and co.), or pre-packed columns and reagent grade solvents.

Materials. All commercial reagents and solvents were used without further purification.

Instrumentation. All ^{1}H NMR, ^{13}C DEPTQ-135 NMR, ^{13}C CPD NMR and ^{19}F NMR spectra were recorded using a Bruker AV-400, AV-500, AV-600 or AV-700 spectrometer at 300 K. Chemical shifts (δ) were given in parts per million (ppm), referenced to the solvent peak of $CDCl_3$, defined at δ = 7.26 ppm (^{1}H NMR) and δ = 77.16 ppm (^{13}C NMR) and the solvent peak of DMSO-d_6, defined at δ = 2.50 ppm (^{1}H NMR) and δ = 39.52 ppm (^{13}C NMR)[1]. Coupling constants (J) are reported in Hertz (Hz). ^{1}H NMR splitting patterns are designated as singlet (s), doublet (d), triplet (t), quartet (q), quintet (quint.) or a combination thereof, as they appeared in the spectrum. If the appearance of a signal differs from the expected splitting pattern, the observed pattern is designated as apparent (app). Splitting patterns that could not be interpreted or easily visualized are designated as multiplet (m) or broad (br). Infrared (IR) spectra were obtained using Perkin-Elmer Spectrum 100 FT-IR spectrometer. Wavenumbers (ν_{max}) are reported in cm^{-1}. Mass spectra were obtained using a Bruker maXis UHR-TOF spectrometer (70 eV), using electrospray ionization (ESI) or atmospheric-pressure chemical

ionization (APCI) or an Agilent 7200B GC/Q-TOF spectrometer (70 eV), using electron ionization (EI). Optical rotations were measured on a Perkin Elmer 341 polarimeter using a 100 mm path-length cell at 589 nm (*c* given in g / (100 mL)). Details of chromatographic conditions are indicated under each compound.

Synthesis of DiSPASO

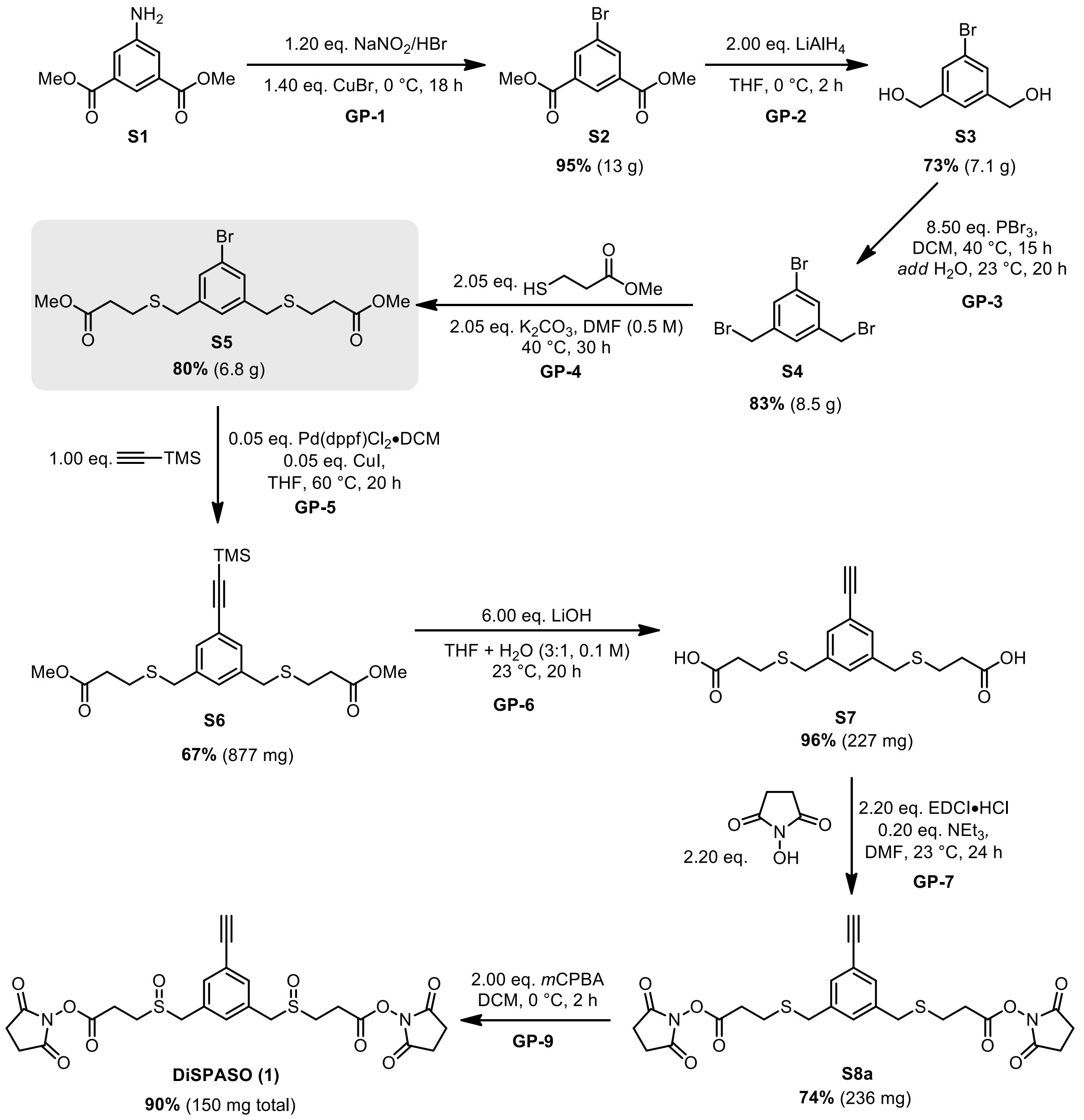

Scheme 1: *DiSPASO synthetic route.*

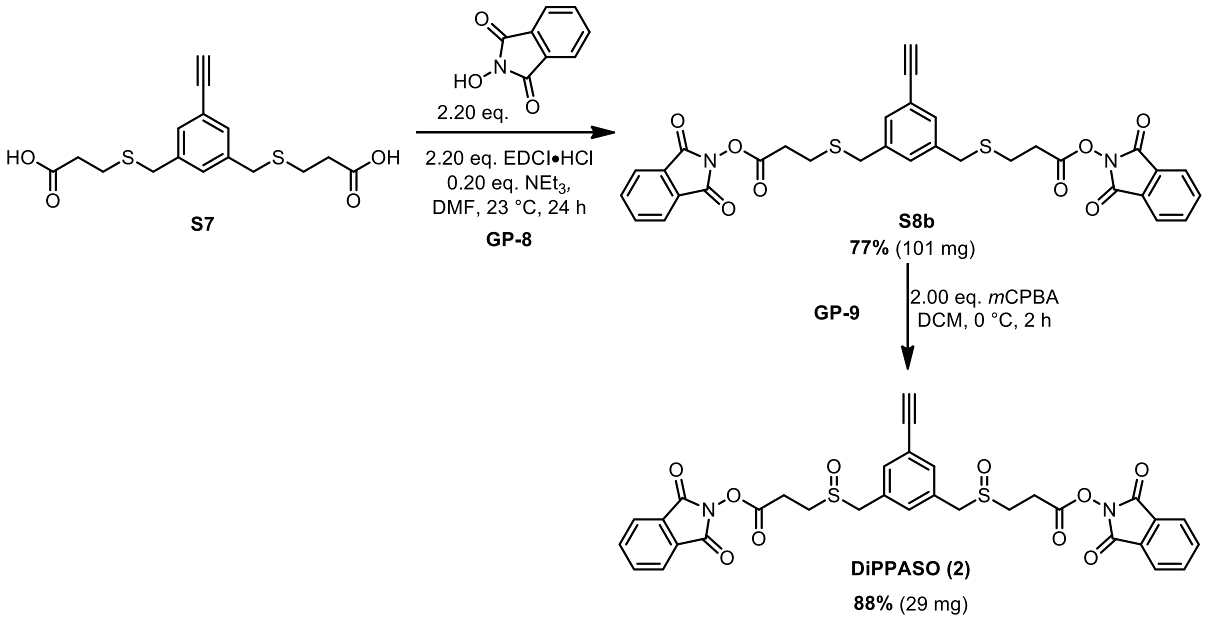

Scheme 2: *DiPPASO synthetic route from common precursor* ***S7****.*

General Procedure 1: Synthesis of Aryl Bromide (GP-1)[2,3]

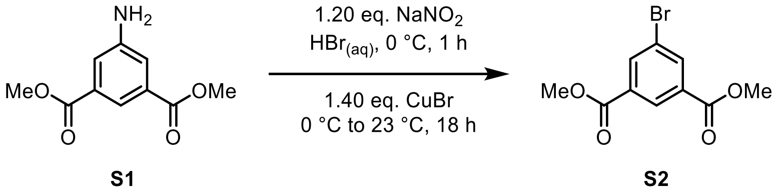

5-Amino-isophthalic acid dimethyl ester (**S1**) (1.00 eq., 50.0 mmol, 10.5 g) was added to a 1 L flask equipped with a magnetic stir bar and dissolved in 200 mL of a 15% aq. HBr solution (obtained by diluting 95 mL of a 48% aq. HBr solution with 205 mL water) at 0 °C. Then an aq. solution of $NaNO_2$ (1.20 eq., 60.0 mmol, 4.14 g in 20.0 mL dist. H_2O) was slowly added under vigorous stirring. After 10 minutes, the diazonium solution was added portion-wise at 0 °C to 1 L flask equipped with a stir bar containing a solution of CuBr (1.40 eq., 70.0 mmol, 10.0 g in 80.0 mL 15% aq. HBr solution) and the reaction mixture was allowed to warm up to 23 °C over 18 h. Then, the reaction mixture was diluted with ethyl acetate (200 mL) and the organic layer was separated, washed with distilled water (3 × 200 mL), dried with $MgSO_4$, filtered and concentrated under reduced pressure. The crude residue was used in the next step without further purification.

Dimethyl 5-bromoisophthalate (**S2**)

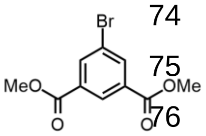

S2

According to general procedure **GP-1**, **S2** was isolated as a yellow solid (12.9 g, 47.2 mmol, 95%) and used in the next step without further purification. Spectroscopic data were in accordance with those reported in the literature[3].

^{1}H NMR (400 MHz, $CDCl_3$): *δ* 8.60 (s, 1H), 8.35 (d, *J* = 1.3 Hz, 2H), 3.96 (s, 6H) ppm.

General Procedure 2: Reduction of Esters (GP-2)

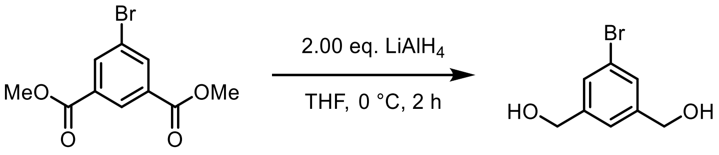

S2 **S3**

To a 500 mL flask equipped with a magnetic stir bar and containing **S2** (1.00 eq., 45.0 mmol, 12.3 g) dissolved in THF (150 mL), were added $LiAlH_4$ pellets (2.00 eq., 90.0 mmol, 3.42 g) at 0 °C. The reaction was stirred at 0 °C for 2 h before being quenched by dropwise addition of sat. potassium sodium tartrate solution (100 mL). The organic phase was separated and the aqueous phase was extracted with diethyl ether (2 × 100 mL). The combined organic phases were dried with $MgSO_4$, filtered and concentrated under reduced pressure. The crude residue was purified by column chromatography.

(5-Bromo-1,3-phenylene)dimethanol (**S3**)

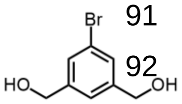

S3

According to general procedure **GP-2**, **S3** was isolated as a white solid (7.14 g, 32.9 mmol, 73%) after purification by column chromatography (DCM/MeOH = 19:1, R_f = 0.29). Spectroscopic data were in accordance with those reported in the literature[3].

^{1}H NMR (400 MHz, $CDCl_3$): *δ* 7.45 (s, 2H), 7.29 (s, 1H), 4.69 (d, *J* = 5.9 Hz, 4H), 1.71 (t, *J* = 5.9 Hz, 2H) ppm.

General Procedure 3: Halogenation of Alcohols (GP-3)

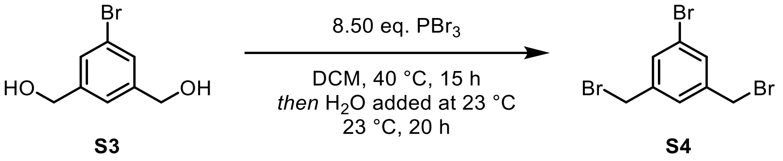

To a 250 mL flame-dried flask equipped with a magnetic stir bar and fitted with a reflux condenser was added **S3**. (1.00 eq., 30.0 mmol, 6.5 g) and dry DCM (100 mL). A solution of PBr_3 (8.50 eq., 255 mmol, 24.7 mL) in dry DCM (100 mL) was added dropwise and the reaction mixture was stirred under reflux (oil bath at 40 °C) for 15 h. Then, the top of the reflux condenser was connected to a series of washing bottles (empty, 1 M NaOH solution, empty) and water (60.0 mL) was added. The reaction was stirred for 20 h at 23 °C and diluted with DCM (30.0 mL). The organic phase was separated. Then the aqueous phase was extracted with DCM (2 × 150 mL). The combined organic phases were dried with $MgSO_4$, filtered and concentrated under reduced pressure. The crude residue was purified by column chromatography.

1-Bromo-3,5-bis(bromomethyl)benzene (**S4**)

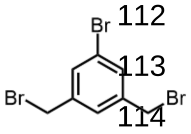

S4

According to general procedure **GP-3**, **S4** was isolated as a white solid (8.52 g, 24.9 mmol, 83%) after purification by column chromatography (heptane/ethyl acetate= 4:1, R_f = 0.35). Spectroscopic data were in accordance with those reported in the literature[3].

^{1}H NMR (400 MHz, $CDCl_3$): δ 7.47 (s, 2H), 7.33 (d, J = 4.3 Hz, 1H), 4.41 (s, 4H) ppm.

^{13}C NMR (101 MHz, $CDCl_3$): δ 140.5, 132.1, 128.4, 122.9, 31.6 ppm.

LRMS (EI^+): exact mass calculated for $[M+Na]^+$ ($C_8H_7Br_3Na$) requires m/z 341.8, found m/z 341.8.

IR (neat) ν_{max}: = 3056, 3024, 2970, 2362, 2106, 1796, 1602, 1571, 1444, 1261, 1210, 1162, 1128, 1114, 998, 972, 893, 878, 865, 819, 740 cm^{-1}.

General Procedure 4: Synthesis of Key Intermediate S5 (GP-4)

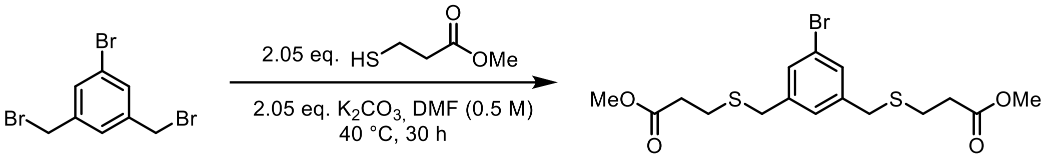

S4 **S5**

To a 250 mL flame-dried flask equipped with a magnetic stir bar and reflux condenser, was added the tribromide **S4** (1.00 eq., 20.0 mmol, 6.86 g), methyl 3-mercaptopropanoate (2.05 eq., 41.0 mmol, 4.66 mL), K_2CO_3 (2.05 eq., 41.0 mmol, 5.67 g) and DMF (40.0 mL). The reaction mixture was heated to 40 °C for 30 h, then the reaction mixture was cooled down to 23 °C and diethyl ether was added (100 mL) and transferred to a separation funnel. The organic phase was washed with H_2O (4 × 100 mL), then brine (3 × 100 mL), dried with $MgSO_4$, filtered, and concentrated under reduced pressure. The crude product was purified by column chromatography.

Dimethyl 3,3'-(((5-bromo-1,3-phenylene)bis(methylene))bis(sulfanediyl))dipropionate (**S5**)

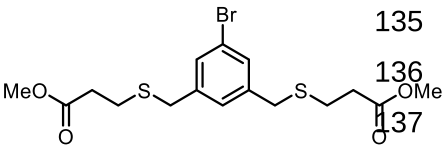

S5

According to general procedure **GP-4**, **S5** was isolated as a colourless oil (6.8 g, 16.1 mmol, 80%) after purification by column chromatography (heptane/ethyl acetate= 9:1 to 5:1, R_f = 0.34 in 5:1).

^{1}H NMR (400 MHz, CDCl$_3$): δ 7.36 (d, J = 1.0 Hz, 2H), 7.21 (s, 1H), 3.69 (s, 6H), 3.67 (s, 4H), 2.69 (dd, J = 10.9, 3.7 Hz, 4H), 2.56 (t, J = 7.1 Hz, 4H) ppm.

^{13}C NMR (101 MHz, CDCl$_3$): δ 172.3 (2C), 140.8 (2C), 130.7 (2C), 128.1, 122.8, 52.0 (2C), 35.9 (2C), 34.4 (2C), 26.5 (2C) ppm.

HRMS (ESI$^+$): exact mass calculated for $[M+Na]^+$ ($C_{16}H_{21}O_4BrNa$) requires *m/z* 442.9957, found *m/z* 442.9957.

IR (neat) **ν_{max}:** = 2996, 2950, 2923, 2844, 1730, 1600, 1568, 1435, 1356, 1299, 1282, 2245, 1217, 1195, 1169, 1018, 979, 946, 893, 865, 820, 728 cm^{-1}.

General Procedure 5: Sonogashira Cross-Coupling (GP-5)

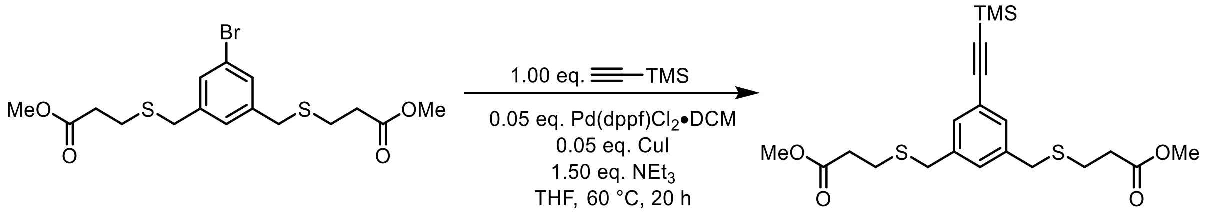

S5 S6

Based on a previously reported procedure[4]. To a flame-dried Schlenk equipped with a magnetic stir bar were added aryl bromide **S5** (1.00 eq., 3.00 mmol, 1.26 g), Pd(dppf)Cl_2•DCM (0.05 eq., 0.15 mmol, 125 mg), CuI (0.05 eq., 0.15 mmol, 28.7 mg) and dry THF (10.0 mL) under inert atmosphere. The reaction mixture was degassed for 20 minutes using an Ar-filled balloon and an ultrasound bath. Then, trimethylsilylacetylene (1.00 eq., 3.00 mmol, 0.43 mL) and NEt_3 (1.50 eq., 4.50 mmol, 0.63 mL) were added and the sealed reaction mixture was stirred for 20 h at 60 °C. Next, the reaction mixture was filtered over a short pad of celite, washed with diethyl ether (20.0 mL), dried with $MgSO_4$, filtered and concentrated under reduced pressure. The crude residue was purified by column chromatography.

Dimethyl 3,3'-(((5-((trimethylsilyl)ethynyl)-1,3-phenylene)bis(methylene))bis(sulfanediyl))-dipropionate (**S6**)

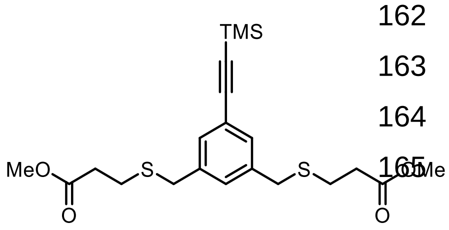

S6

According to general procedure **GP-5**[4], **S6** was isolated as a colourless oil (877 mg g, 2.00 mmol, 67%) after purification by column chromatography (heptane/ethyl acetate= 9:1, R_f = 0.21)

^{1}H NMR (400 MHz, $CDCl_3$): *δ* 7.30 (s, 2H), 7.24 (s, 1H), 3.68 (d, *J* = 0.5 Hz, 6H), 3.67 (s, 4H), 2.66 (t, *J* = 7.2 Hz, 4H), 2.55 (t, *J* = 7.2 Hz, 4H), 0.24 (s, 9H) ppm.

^{13}C NMR (101 MHz, $CDCl_3$): *δ* 172.3 (2C), 138.8, 131.2 (2C), 129.7, 123.7, 104.6, 94.8, 51.9 (2C), 36.0 (2C), 34.4 (2C), 26.4 (2C), 0.1 (3C) ppm.

HRMS (ESI$^+$): exact mass calculated for $[M+H]^+$ ($C_{21}H_{31}O_4S_2Si$) requires *m/z* 439.1428, found *m/z* 439.1428.

IR (neat) ν_{max}: = 2953, 2159, 1734, 1593, 1435, 1357, 1299, 1248, 1196, 1168, 1020, 980, 840, 759 cm^{-1}.

General Procedure 6: Global Deprotection (GP-6)

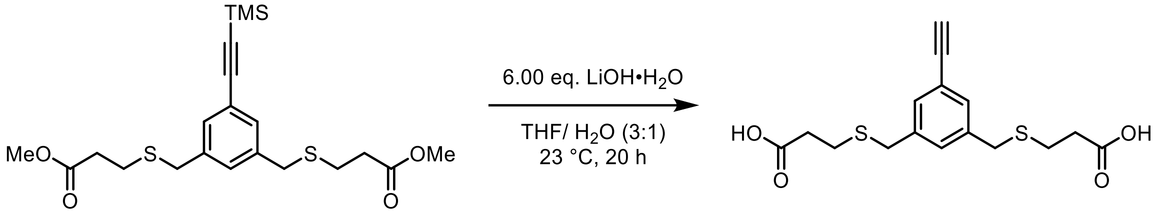

S6 **S7**

In a flame-dried flask equipped with a magnetic stir bar **S6** (1.00 eq., 0.70 mmol, 307 mg) was dissolved in a THF/H_2O mixture (3:1 *v*/*v*, 4.0 mL). LiOH•H_2O (6.00 eq., 4.20 mmol, 176 mg) was added and the reaction was stirred at 23 °C for 24 h. The reaction was stopped by the addition of a 1 M HCl solution (5.0 mL) and the mixture was transferred to a separation funnel. The organic phase was separated, then the aqueous phase was washed with diethyl ether (4 × 5.0 mL). The combined organic phase was dried with $MgSO_4$, filtered and concentrated under reduced pressure. The crude residue was used for the next step without further purification.

3,3'-(((5-Ethynyl-1,3-phenylene)bis(methylene))bis(sulfanediyl))dipropionic acid (**S7**)

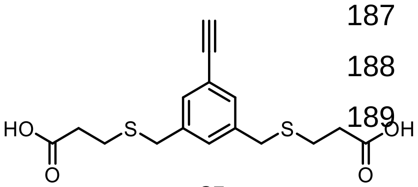

S7

According to general procedure **GP-6, S7** was isolated as a white solid (227 mg, 0.67 mmol, 96%) and used for the next step without further purification.

^{1}H NMR (500 MHz, $CDCl_3$): *δ* 7.35 (d, *J* = 1.2 Hz, 2H), 7.29 (s, 1H), 3.71 (s, 4H), 3.07 (s, 1H), 2.68 (t, *J* = 6.9 Hz, 4H), 2.58 (t, *J* = 6.9 Hz, 4H) ppm.

^{13}C NMR (151 MHz, $CDCl_3$): *δ* 177.9 (2C), 138.9 (2C), 131.4 (2C), 130.1, 122.9, 77.8, 68.1, 36.1 (2C), 34.5 (2C), 26.0 (2C) ppm.

HRMS (ESI$^-$): exact mass calculated for [M-H]$^-$ ($C_{16}H_{17}O_4S_2$) requires *m/z* 337.0574, found *m/z* 337.0575.

IR (neat) v_{max}: = 3231, 2916, 2664, 1688, 1590, 1420, 1403, 1337, 1302, 1264, 1237, 1197, 1161, 1141, 1041, 910, 880, 869, 805, 771 cm^{-1}.

General Procedure 7: Synthesis of NHS Protected Precursor S8 (GP-7)

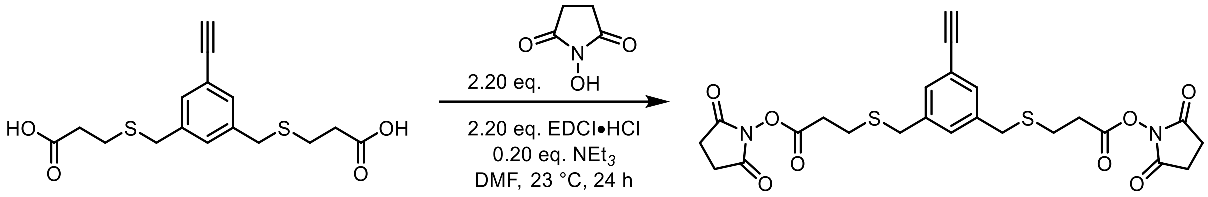

S7 **S8a**

Inspired by previously reported procedure[5]. To flame-dried Schlenk equipped with a stir bar was added the diacid **S7** (1.00 eq., 0.60 mmol, 203 mg) and dry DMF (6.0 mL) under an inert atmosphere. Next, *N*-hydroxysuccinimide (2.20 eq., 1.32 mmol, 155 mg), 1-(3-dimethylaminopropyl)-3-ethylcarbodiimide hydrochloride (EDCI•HCl, 2.20 eq., 1.32 mmol, 253 mg) and NEt_3 (0.20 eq., 0.12 mmol, 17.0 µL) were sequentially added at 23 °C. The reaction mixture was stirred at 23 °C for 14 h. Then the reaction mixture was stopped by the addition of an aq. 1 M HCl solution (10.0 mL), diluted with ethyl acetate (10.0 mL) and transferred to a separation funnel. The organic phase was separated, and washed with sat. ammonium chloride solution (2 × 10.0 mL), distilled H_2O (2 × 10.0 mL) and brine (2 × 10.0 mL), dried with $MgSO_4$, filtered and concentrated under reduced pressure. The crude residue was purified by column chromatography.

Bis(2,5-dioxopyrrolidin-1-yl)3,3'-(((5-ethynyl-1,3-phenylene)bis(methylene)) bis(sulfanediyl))dipropionate (**S8a**)

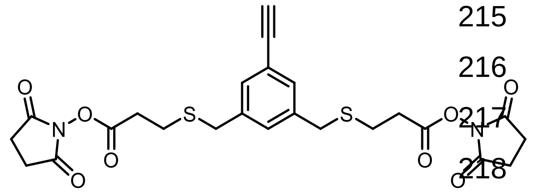

S8a

According to general procedure **GP-7**[5], **S8** was isolated as a colourless oil (236 mg, 0.44 mmol, 74%) after purification by column chromatography (heptane/ethyl acetate= 1:2, R_f = 0.28).

^{1}H NMR (400 MHz, CDCl$_3$): δ 7.36 (d, J = 1.2 Hz, 2H), 7.31 (s, 1H), 3.73 (s, 4H), 3.08 (s, 1H), 2.87 – 2.78 (m, 12H), 2.79 – 2.71 (m, 4H) ppm.

^{13}C NMR (151 MHz, CDCl$_3$): δ 169.1 (4C), 167.2 (2C), 138.8 (2C), 131.5 (2C), 130.1, 123.0, 83.2, 77.8, 36.0 (2C), 31.8 (2C), 25.8 (2C), 25.7 (4C) ppm.

HRMS (ESI$^+$): exact mass calculated for $[M+Na]^+$ ($C_{24}H_{24}N_2O_8S_2Na$) requires *m/z* 555.0866, found *m/z* 555.0868.

IR (neat) ν_{max}: = 3232, 2916, 2664, 1688, 1590, 1420, 1403, 1337, 1302, 1264, 1237, 1197, 1161, 1141, 1041, 910, 880, 869, 805, 771 cm^{-1}.

General Procedure 8: Synthesis of NHP Protected Precursor (GP-8)

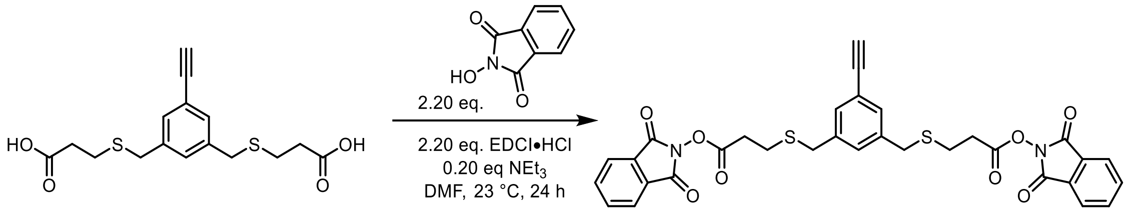

S7 S8b

To flame-dried Schlenk equipped with a stir bar was added diacid **S7** (1.00 eq., 0.21 mmol, 71 mg) and dry DMF (2.0 mL) under an inert atmosphere. Then, *N*-hydroxyphthalimide (2.20 eq., 0.46 mmol, 75 mg), 1-(3-dimethylaminopropyl)-3-ethylcarbodiimide hydrochloride (EDCI•HCl, 2.20 eq., 0.46 mmol, 88 mg) and NEt_3 (0.20 eq., 0.04 mmol, 6.0 µL) were added sequentially at 23 °C. The reaction mixture was stirred at 23 °C for 14 h before being quenched by the addition of aq. 1 M HCl (3.0 mL), diluted with ethyl acetate (3.0 mL) and transferred to a separation funnel. The organic phase was separated, and washed with sat. ammonium chloride solution (2 × 3.0 mL), distilled H_2O (2 × 3.0 mL) and brine (2 × 3.0 mL), dried with $MgSO_4$, filtered, and concentrated under reduced pressure. The crude residue was purified by column chromatography.

(Bis(1,3-dioxoisoindolin-2-yl) 3,3'-(((5-ethynyl-1,3-phenylene)bis(methylene))bis(sulfanediyl))dipropionate (**S8b**)

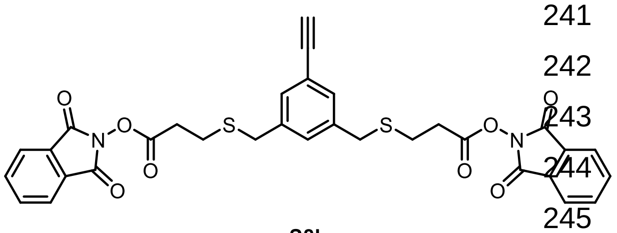

S8b

According to general procedure **GP-8**, **S8b** was isolated as a white solid (101 mg, 0.16 mmol, 77%) after purification by column chromatography (heptane/ethyl acetate = 9:1, R_f = 0.27).

^{1}H NMR (400 MHz, CDCl$_3$): *δ* 7.93 – 7.84 (m, 4H), 7.83 – 7.75 (m, 4H), 7.39 (d, *J* = 1.5 Hz, 2H), 7.36 (s, 1H), 3.77 (s, 4H), 3.06 (s, 1H), 2.92 (dd, *J* = 11.0, 3.9 Hz, 4H), 2.81 (dd, *J* = 11.2, 4.2 Hz, 4H) ppm.

^{13}C NMR (101 MHz, CDCl$_3$): *δ* 168.2 (2C), 161.9 (4C), 138.8 (2C), 135.0 (4C), 131.6 (2C), 130.2, 129.0 (4C), 124.2 (4C), 123.0, 83.2, 77.8, 36.1 (2C), 31.9 (2C), 26.0 (2C) ppm.

HRMS (ESI$^+$): The exact mass calculated for $[M+Na]^+$ ($C_{32}H_{24}N_2O_8S_2Na$) requires *m/z* 651.0866, found *m/z* 651.0868.

IR (neat) v_{max}: = 3274, 2924, 2853, 1815, 1786, 1738, 1594, 1467, 1450, 1412, 1358, 1325, 1290, 1267, 1241, 1184, 1134, 1078, 1034, 963, 877, 830, 786 cm^{-1}.

<u>General Procedure 9: Synthesis of DiSPASO and DiPPASO (GP-9)</u>

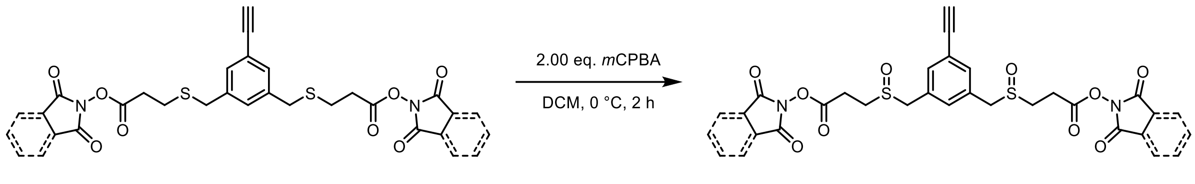

S8a or **S8b** **1** or **2**

To flame-dried 4 mL vial equipped with a magnetic stir bar was added the corresponding precursor **S8a** or **S8b** (1.00 eq., 0.05 mmol) and dry DCM (0.5 mL) under an inert atmosphere. Next, *m*CPBA (2.00 eq.) dissolved in 0.5 mL of dry DCM was added dropwise at 0 °C. The reaction mixture was stirred at 0 °C for 2 h followed by the addition of 3.0 mL of sat. aq. $NaHCO_3$ solution and 3.0 mL DCM. The organic phase was separated, and washed with sat. aq. $NaHCO_3$ solution (2 × 3.0 mL), distilled H_2O (3.0 mL) and brine (3.0 mL), dried with $MgSO_4$, filtered, and concentrated under reduced pressure. The crude product was stored in a vial, under an inert atmosphere at –20 °C.

<u>DiSPASO (1)</u>

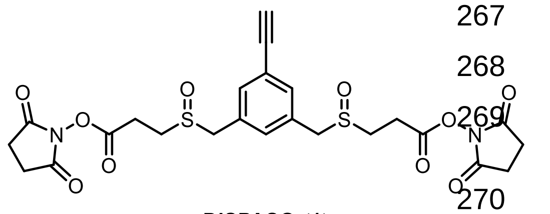

DiSPASO (**1**)

According to general procedure **GP-9**, **1** was isolated as a white solid (25.5 mg, 0.05 mmol, 90%).

^{1}H NMR (600 MHz, $CDCl_3$): δ 7.45 (s, 2H), 7.26 (s, 1H, confirmed by HMBC), 4.0 (s, 4H), 3.20 – 3.03 (m, 7H), 2.93 – 2.87 (m, 2H), 2.85 (s, 8H) ppm.

^{13}C NMR (151 MHz, $CDCl_3$): δ 168.9 (4C), 167.2, 167.2, 133.8, 133.8, 132.2 (2C), 132.2 (2C), 130.9, 130.8, 124.2, 82.0 (observed in HMBC), 79.4, 57.6, 57.6, 44.8 (2C), 25.7 (4C), 24.2, 24.2 ppm.

HRMS (ESI$^+$): The exact mass calculated for $[M+Na]^+$ ($C_{24}H_{24}N_2O_{10}S_2Na$) requires *m/z* 587.0765, found *m/z* 587.0765.

IR (neat) v_{max}: = 3270, 2923, 2853, 2359, 1812, 1782, 1735, 1595, 1429, 1367, 1207, 1090, 1046, 894, 744 cm^{-1}.

DiPPASO(Bis(1,3-dioxoisoindolin-2-yl) 3,3'-((5-ethynyl-1,3-phenylene)bis(methylenesulfinyl))dipro-pionate, **2**)

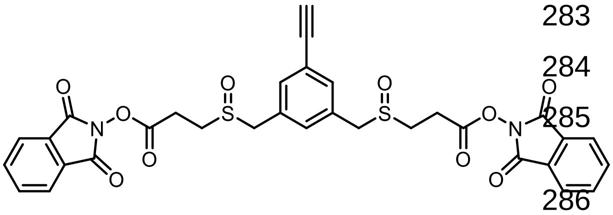

DiPPASO (**2**)

According to general procedure **GP-9**, **2** was isolated as a white solid (29.1 mg, 0.04 mmol, 88%).

^{1}H NMR (400 MHz, CDCl$_3$): δ 7.89 (dt, J = 7.1, 3.6 Hz, 4H), 7.84 – 7.76 (m, 4H), 7.47 (d, J = 1.1 Hz, 2H), 7.30 (s, 1H), 4.02 (s, 4H), 3.28 – 2.89 (m, 9H) ppm.

^{13}C NMR (176 MHz, CDCl$_3$): δ 168.2, 168.2, 161.7 (4C), 135.1 (4C), 133.9 (2C), 132.3, 132.3, 130.9 (2C), 128.9 (2C), 124.3 (4C), 124.2 (2C), 79.4, 77.4, 57.7 (2C), 57.7 (2C), 45.1 (2C), 45.0 (2C), 24.2 (2C), 24.2 (2C) ppm.

HRMS (ESI$^+$): The exact mass calculated for [M+Na]$^+$ ($C_{32}H_{24}N_2O_{10}S_2Na$) requires m/z 683.0765, found m/z 683.0765.

IR (neat) v_{max}: = 3276, 2924, 2854, 1815. 1787, 1740, 1596, 1467, 1413, 1361, 1267, 1186, 1157, 1136, 1083, 1040, 964, 877, 824, 787 cm^{-1}.

Supplemental figures

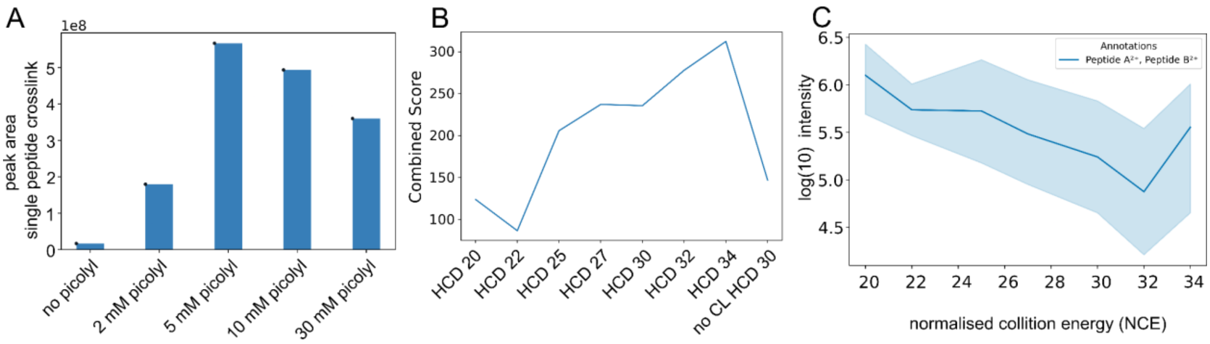

Figure S1: Single peptide evaluation of DiSPASO. A: Titration experiment of a single Peptide crosslinked with DiSPASO. The picolyl azide concentrations were set in increasing order to 2 mM, 5 mM, 10 mM and 30 mM. The non-crosslinked peptide was used as a control. Increasing the amount of picolyl azide also increased the peak area of the crosslinked product with a maximum of 5 mM compound. B: Titration series of high-performance collision energies concerning the combined score of peptides a and b within the crosslink. The score of a crosslinked peptide increases with high energies with a maximum at HCD 34. C: Log(10) intensities of the 32 Da fragment doublet pair after fragmentation of DiSPASO crosslinked single peptide. The intensity of the doublet decreases with higher energies but rises again with HCD 34.

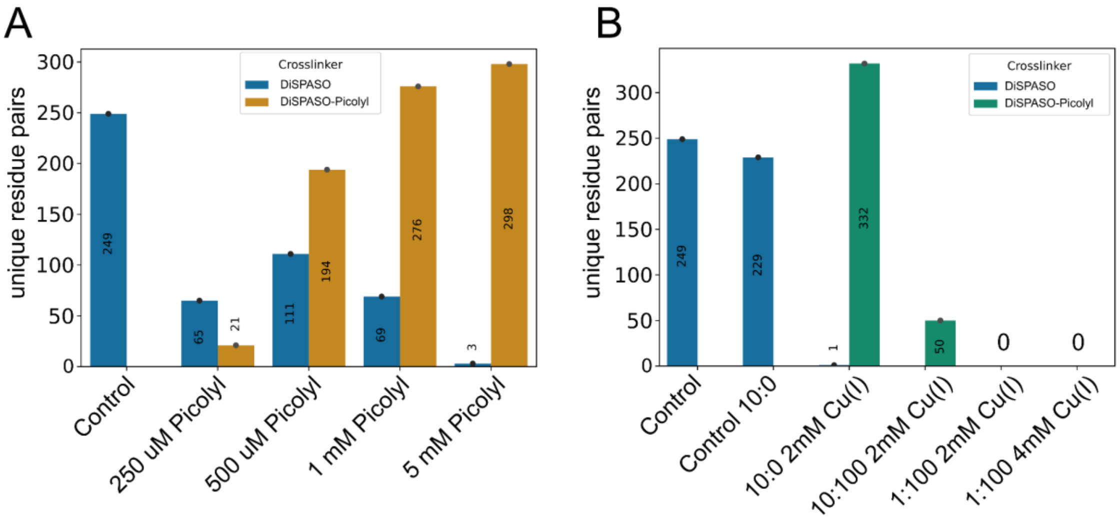

Figure S2: Evaluation of picolyl concentration for click reaction and enrichment sensitivity in Cas9 only and with HeLa background. A: Picolyl azide concentration titration using crosslinked Cas9 with DiSPASO. Non-enriched DiSPASO crosslinked peptides were used as the control sample (blue), whereas the titration series with increasing amounts of picolyl azide is shown in yellow. The maximum number of identified crosslinked peptides could be achieved with 5 mM picolyl azide as already shown in Figure S1A with single peptide crosslinking. B: Challenging the picolyl azide enrichment with HeLa background. When Cas9 crosslinked with DiSPASO is spiked into HeLa background (10:100 and 1:100) crosslinked peptides can't be enriched sufficiently anymore. The numbers drop from a 10:0 ratio (not spiked-in) to 10:100 with a 100ug HEK lysate background. Crosslinks are not identifiable anymore at in-cell crosslinking levels of 1:100.

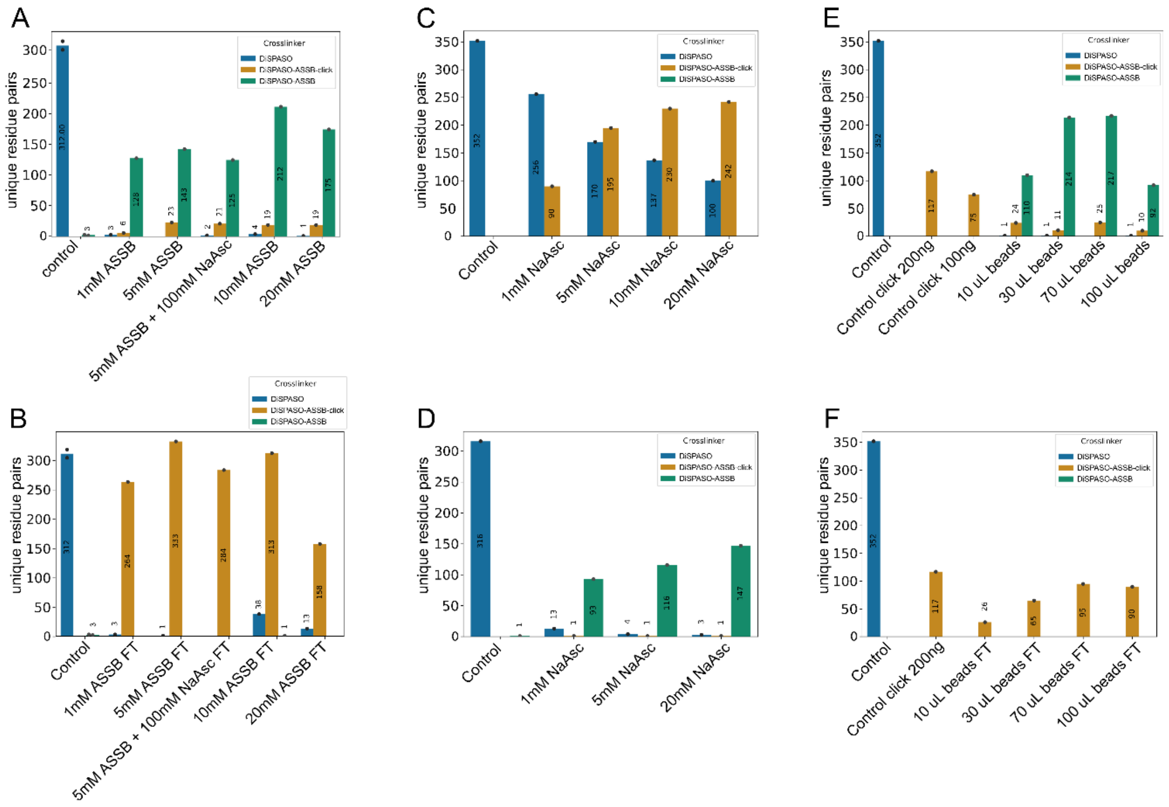

Figure S3: Optimization of Azide-S-S-biotin, sodium ascorbate and bead amount to achieve optimal click and enrichment performance. A: Titration of the optimal Azide-S-S-biotin (ASSB) amount to achieve high click reaction performance. The concentration was set to 1 mM, 5 mM, 5 mM + 100 mM sodium ascorbate, 10 mM and 20 mM. After 20 mM the maximum solubility in the reaction mix is reached. At 10 mM ASSB, the total number of identified residue pairs reaches its maximum. B: The flowthrough of this experiment was also assessed to check for potential losses during the workflow. The overall loss seems to be independent of the ASSB concentration and is in general high. C: Titration of the optimal sodium ascorbate (NaAsc) concentration for click reaction. The concentration was set to 1 mM, 5 mM, 10 mM and 20 mM. After 10 mM NaAsc, the enrichment performance reaches almost its plateau. The click reaction efficiency can be estimated by following the decrease of non-clicked DiSPASO crosslinked peptides (blue) to the increase of click product after click reaction (yellow). D: Performance of biotin-streptavidin bead enrichment after ASSB optimization. The optimal enrichment performance could be achieved with 10 mM ASSB and 20 mM Sodium ascorbate. The NaAsc concentration was set to 30 mM for further experiment to ensure high click performance while reducing potential side reaction of the copper-based click reaction. E: To reduce the loss of the bead-based enrichment strategy a titration of the right bead amount was performed. The volume of the beads was set to 10 uL, 30 uL, 70 uL and 100 uL of MBS bead slurry. Most residue pairs could be identified with 70 uL beads for enrichment. F: The flowthrough after bead enrichment was tested again to assess the loss after bead optimization, the loss could be reduced by half but is still present even after using 100 uL of beads.

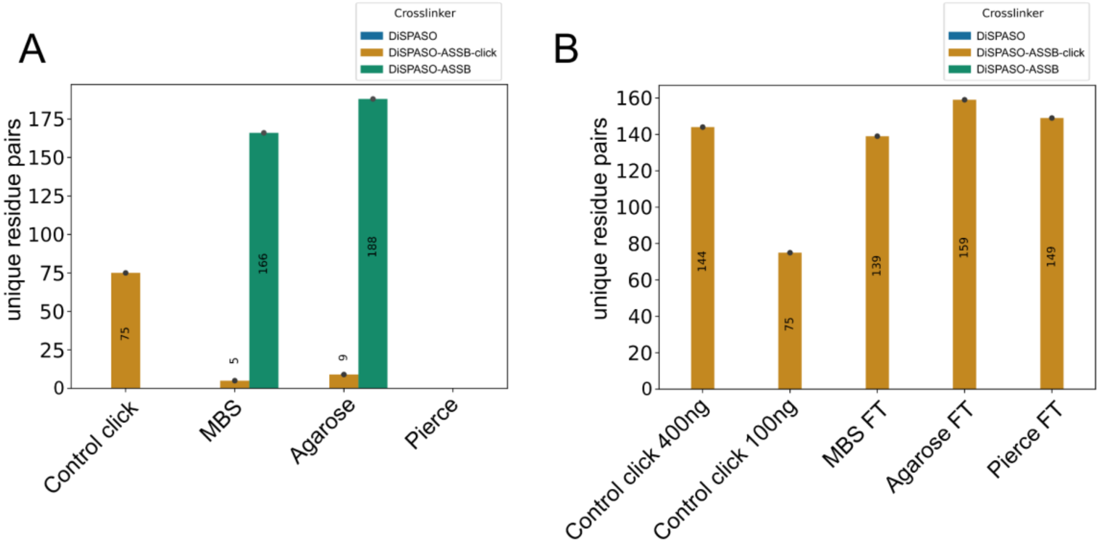

Figure S4: Comparison of different bead types. A: Different bead types were tested to ensure that the loss of crosslinks after enrichment is not caused by the beads. Three types of beads have been tested, Pierce™ High-Capacity Streptavidin Agarose (Agarose), Pierce™ Streptavidin Magnetic Beads (Pierce) and MBS Magnetic beads-streptavidin (MBS) with Agarose showing the most identified crosslinks after bead enrichment. B: Flowthrough of the experiment showing a high loss of crosslinked peptides after enrichment. The loss of crosslinked peptides might also be independent of the bead type.

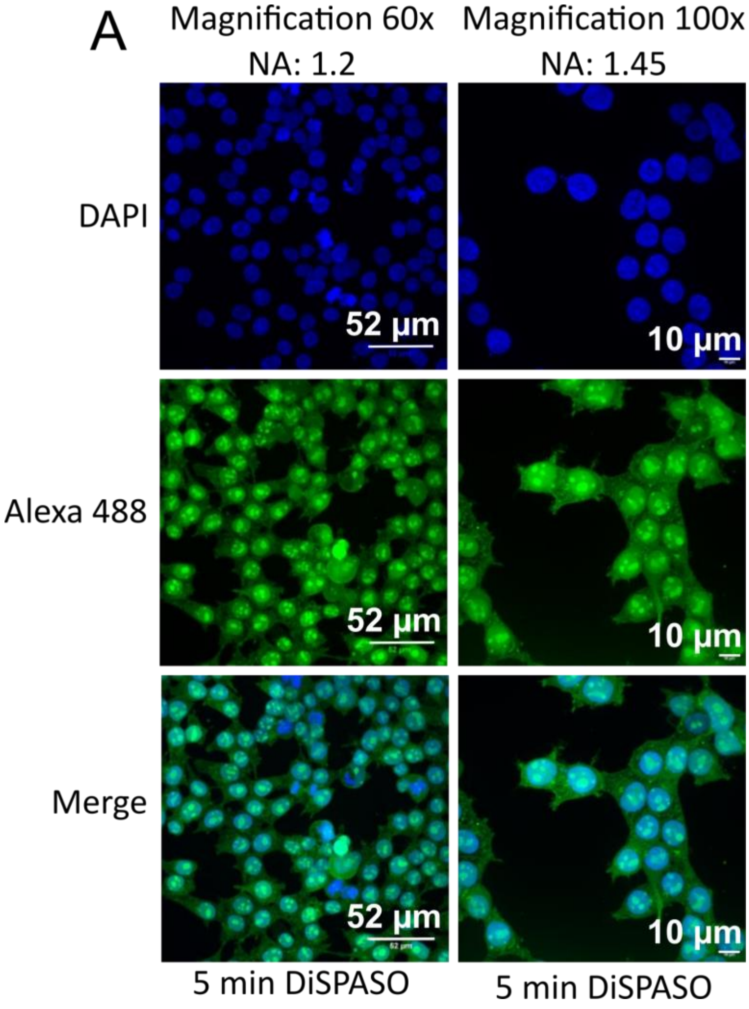

Figure S5: Confocal microscopy pictures of crosslinked HEK 293 cells using DiSPASO. A: Confocal microscopy images of DiSPASO during in-cell crosslinking experiments with a crosslink duration of 5min. The nuclei fluorescence signal of DAPI is shown in the upper panel in blue, fluorescence of crosslinked peptides after click reaction to Alexa 488 (green) in the middle panel and a merge of both channels on the bottom. The images were taken on an Olympus Spinning Disk Confocal microscope (2-024) using a magnification of either 60 and a numerical aperture of 1.2 (left panel) or a numerical 100 and a numerical aperture of 1.45 (right panel).

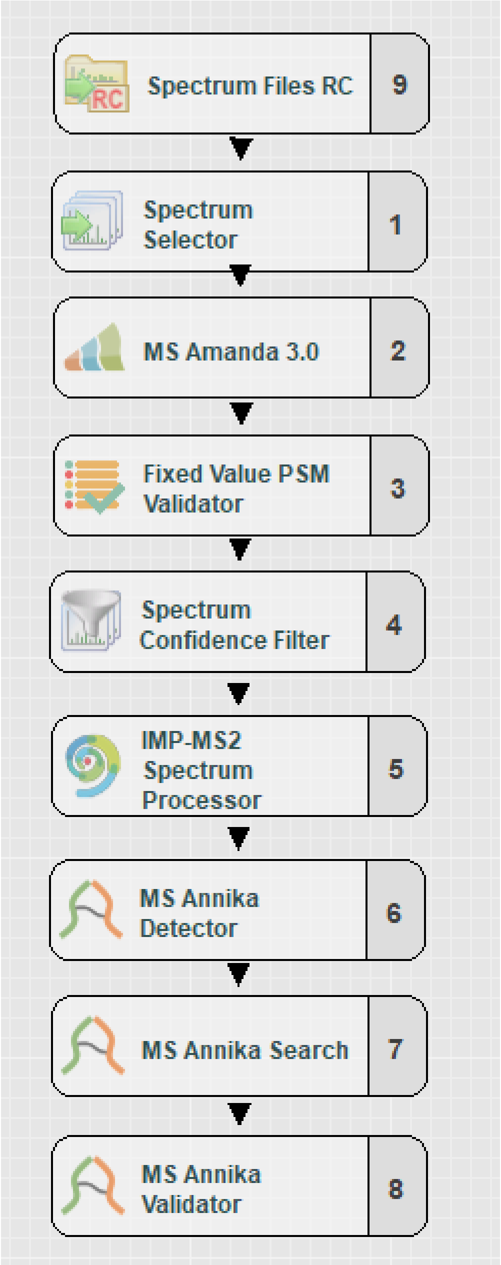

Figure S6: Exemplary workflow of an MS/MS2 search with MS Annika 2.0 in Proteome Discoverer. The imported raw files are recalibrated by the Spectrum file RC node, followed by a crude selection of suitable spectra for a first search by MS Amanda for linear peptide identification. Spectra that could not map to a linear or monolink peptide are transferred to the MS Annika crosslink search and validation nodes. The crosslink doublet peaks are deisotoped for calculating the peptide's monoisotopic mass, and spectra are adjusted and then searched with MS Annika to identify the cross-linked peptides.

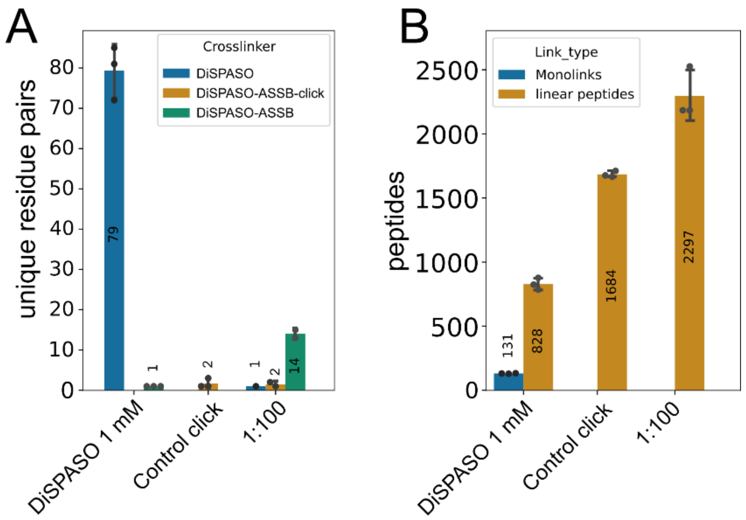

Figure S7: Application of ASSB-DiSPASO enrichment strategies of spike-in ribosome samples. A: Crosslinked E. coli (K12) ribosome spike-in with HEK 293 cell lysate as background. The ribosome spike-in increased from 0.25ug to 10ug in a constant background of 100ug HEK lysate (only the 1:100 mix is shown here). Part of the 10:100 sample was used as a control (Control-click), showing the performance of ribosome crosslinking without enrichment. The second control sample shows the overall performance of ribosome crosslinking without click reaction or enrichment (blue). The enrichment of 1ug ribosome crosslinked in a 100ug HEK 293 cell lysate background is shown in green. B: Analysis of monolinks and linear peptides of the ribosome spike-in experiment. The monolinks and linear peptides of the "crosslink control" sample show fewer peptides due to ribosome-only related linear peptides that can occur here. The HEK background peptides of the spike-in could be not depleted. The number of technical replicates is indicated as separate black dots on top of the bar (n=3). The standard deviation was estimated as average distance from each data point to the sample mean.

Supplemental tables

Table S1. Special reagents used for DiSPASO click-reaction, enrichment, and microscopy.

Reagent name	Catalogue number	Supplier
CuAAC Biomolecule Reaction Buffer Kit (BTTAA-based)	CLK-071	Jena Bioscience
MBS magnetic beads Streptavidin	LOT 19-1	Molecular Biology Services (in-house)
Disulfide Azide Agarose	CLK-CSTM, 1238-2	Click Chemistry Tools
Pierce™ High Capacity Streptavidin Agarose	20359	Thermo Fisher Scientific
Pierce™ Streptavidin Magnetic Beads	88816	Thermo Fisher Scientific
E. coli Ribosome	P0763S	BioLabs

Azide-SS-biotin	BP-22877	BroadPharm
µ-Dish 35 mm, high Grid-500 Glass Bottom	81168	Ibidi
Tris[(1-Benzyl-1H-1,2,3-Triazol-4-yl)methyl]amin	678937	Sigma-Aldrich
Cas9 from *S. pyogenes* fused with a Halo-tag	In house	Deng *et al.*[6]
Trypsin gold	V5280	Promega
Lysyl endopeptidase (LysC)	125-05061	Wako
Human HeLa cells	CCL-2	ATCC
Human HEK 293 cells	CRL-1573	ATCC

Table S2: Fragment names, substitution, and monoisotopic masses of DiSPASO fragments used for crosslinking search.

Fragment name	Substitution	Monoisotopic mass [Da]
DiSPASO	C16H14O4S2	334.0333
Alkene (common for all CLs)	C3H2O	54.01056
Sulfenic acid (common for all CLs)	C3H4O2S	103.9932
Thiol (common for all CLs)	C3H2OS	85.98264
ETFP	C13H10O2S2	262.0122
ETHMP	C13H12O3S2	280.0227
EMP	C13H12O2S	232.055
**Picolyl**		
DiSPASO-Picoly	C27H30N5O8PS2	647.1273
ETFP	C24H26N5O6PS2	575.1062
ETHMP	C24H28N5O7PS2	593.1167
EMP	C24H28N5O6PS	545.1497
**ASSB**		
DiSPASO-ASSB	C20H22N4O5S3	494.0752

ETFP	C17H18N4O3S3	422.0541
ETHMP	C17H20N4O4S3	440.0646
EMP	C17H20N4O3S2	392.0976
**ASSB_click**		
DiSPASO-ASSB_click	C30H38N6O6S5	738.1456
ETFP	C27H34N6O4S5	666.1245
ETHMP	C27H36N6O5S5	684.1350
EMP	C27H36N6O4S4	636.1680
**DAAB**		
DiSPASO-DAAB	C24H31N5O6S3	579.1279972
ETFP	C21H25N5O4S3	507.10687
ETHMP	C21H27N5O5S3	525.11743
EMP	C21H27N5O4S2	477.15045

Table S3: Search parameters for linear and crosslink search. Parameters not listed here were left at default settings

Parameter name	Parameter value
**Linear search**	
MS1 tolerance	6 ppm
MS2 tolerance	15 ppm
Miss cleavages	3
Fixed modification	Carbamidomethyl [57.021 Da]
Variable modification	Oxidation [15.995 Da], DiSPASO amidated [351.059 Da], DiSPASO loop [334.033 Da], DiSPASO hydrolysed [352.043 Da], DiSPASO Tris [454.099 Da], DSBSO amidated [325.065 Da], DSBSO loop [308.038 Da], DSBSO hydrolysed [326.049 Da], DSBSO Tris [429.112 Da], Variable modifications for all other crosslinker definitions were calculated accordingly
Variable modification for in-cell searches	Phospho [79.966 Da], Deamidation [0.984

(additional)	Da], Acetyl Protein N-term [42.011 Da]
**Crosslink search**	
MS1 tolerance	6 ppm
MS2 tolerance	15 ppm
Miss cleavages	3
Fixed modification	Carbamidomethyl [57.021 Da]
Variable modification	Oxidation [15.995 Da]
**IMP-MS2 spectrum processor**	
Perform de-isotoping	False

Table S4: IUPAC and supplier names of chemical compounds and their abbreviations used in this manuscript.

Number of compounds	IUPAC name	Abbreviation
1	bis(2,5-dioxopyrrolidin-1-yl) 3,3'-((5-ethynyl-1,3-phenylene)bis(methylenesulfinyl))dipropanoate	DiSPASO
2	Bis(1,3-dioxoisoindolin-2-yl) 3,3'-((5-ethynyl-1,3-phenylene)bis(methylenesulfinyl))dipro-pionate	DiPPASO
3	(4-(6-(azidomethyl)nicotinamido)butyl)phosphonic acid	Picolyl azide
4	(4-(6-((4-(3,5-bis(((3-((2,5-dioxopyrrolidin-1-yl)oxy)-3-oxopropyl)sulfinyl)methyl)phenyl)-1H-1,2,3-triazol-1-yl)methyl)nicotinamido)butyl)phosphonic acid	BPNB
5	N-(2-((2-azidoethyl)disulfanyl)ethyl)-5-(2-oxohexahydro-1H-thieno[3,4-d]imidazol-4-yl)pentanamide	ASSB
6	2-((2-(4-(3,5-bis(((3-(2,5-dioxopyrrolidin-1-yl)-3-oxopropyl)sulfinyl)methyl)phenyl)-1H-1,2,3-triazol-1-yl)ethyl)thio)acetamide	BAED
7	Disulfide azide beads (supplier name)	DAAB
8	1,1'-(3,3'-((5-(1-(3-aminopropyl)-1H-1,2,3-triazol-4-yl)-1,3-phenylene)bis(methylenesulfinyl))bis(propanoyl))bis(pyrrolidine-2,5-dione)	BAPD
9	3-((3-ethynyl-5-thioformylbenzyl)sulfinyl)propanal	ETFP
10	3-((3-ethynyl-5-((hydroxythio)methyl)benzyl)sulfinyl)propanal	ETHMP
11	Acrylaldehyde (Alkene)	A

12	3-((3-ethynyl-5-methylbenzyl)sulfinyl)propanal	EMP
13	3-(hydroxythio)propanal (Sulfenic acid)	SA
14	(E)-3-mercaptoacrylaldehyde (Thiol)	T

References

1. Fulmer, G. R. *et al.* NMR Chemical Shifts of Trace Impurities: Common Laboratory Solvents, Organics, and Gases in Deuterated Solvents Relevant to the Organometallic Chemist. (2010) doi:10.1021/om100106e.
2. Mazik, M. & König, A. Recognition properties of an acyclic biphenyl-based receptor toward carbohydrates. *J. Org. Chem.* **71**, 7854–7857 (2006).
3. Chen, Z. *et al.* A New Multidentate Hexacarboxylic Acid for the Construction of Porous Metal−Organic Frameworks of Diverse Structures and Porosities. (2010) doi:10.1021/cg100316s.
4. Crosignani, S. *et al.* Discovery of Potent, Selective, and Orally Bioavailable Alkynylphenoxyacetic Acid CRTH2 (DP2) Receptor Antagonists for the Treatment of Allergic Inflammatory Diseases. (2011) doi:10.1021/jm200866y.
5. Marcum, J. S., Taylor, T. R. & Meek, S. J. Enantioselective Synthesis of Functionalized Arenes by Nickel-Catalyzed Site-Selective Hydroarylation of 1,3-Dienes with Aryl Boronates. *Angew. Chem. Int. Ed Engl.* **59**, 14070–14075 (2020).
6. Deng, W., Shi, X., Tjian, R., Lionnet, T. & Singer, R. H. CASFISH: CRISPR/Cas9-mediated in situ labeling of genomic loci in fixed cells. *Proc. Natl. Acad. Sci. U. S. A.* **112**, 11870–11875 (2015).
7. Rappsilber, J., Mann, M. & Ishihama, Y. Protocol for micro-purification, enrichment, pre-fractionation and storage of peptides for proteomics using StageTips. *Nat. Protoc.* **2**, 1896–1906 (2007).
8. Ishihama, Y., Rappsilber, J. & Mann, M. Modular stop and go extraction tips with stacked

disks for parallel and multidimensional peptide fractionation in proteomics. *J. Proteome Res.* **5**, 988–994 (2006).

9. Müller, F., Graziadei, A. & Rappsilber, J. Quantitative photo-crosslinking mass spectrometry reveals protein structure response to environmental changes. *Anal. Chem.* (2019) doi:10.1021/acs.analchem.9b01339.
10. Dorfer, V. *et al.* MS Amanda, a Universal Identification Algorithm Optimized for High Accuracy Tandem Mass Spectra. (2014) doi:10.1021/pr500202e.
11. Pirklbauer, G. J. *et al.* MS Annika: A New Cross-Linking Search Engine. *J. Proteome Res.* (2021) doi:10.1021/acs.jproteome.0c01000.
12. Birklbauer, M. J., Matzinger, M., Müller, F., Mechtler, K. & Dorfer, V. MS Annika 2.0 Identifies Cross-Linked Peptides in MS2–MS3-Based Workflows at High Sensitivity and Specificity. *J. Proteome Res.* (2023) doi:10.1021/acs.jproteome.3c00325.
13. Nelli, F. *Pandas in 7 Days: Utilize Python to Manipulate Data, Conduct Scientific Computing, Time Series Analysis, and Exploratory Data Analysis (English Edition)*. (BPB Publications, 2022).
14. Harris, C. R. *et al.* Array programming with NumPy. *Nature* **585**, 357–362 (2020).
15. Hunter, J. D. Matplotlib: A 2D Graphics Environment. *Comput. Sci. Eng.* **9**, 90–95 (2007).
16. Waskom, M. seaborn: statistical data visualization. *J. Open Source Softw.* **6**, 3021 (2021).
17. Garreta, R. & Moncecchi, G. *Learning Scikit-Learn: Machine Learning in Python*. (Packt Pub Limited, 2013).
18. Virtanen, P. *et al.* SciPy 1.0: fundamental algorithms for scientific computing in Python. *Nat. Methods* **17**, 261–272 (2020).
