# Supplementary material for: Developing a new cleavable crosslinker reagent for in-cell crosslinking
Source: Commun Chem. 2025 Jun 23;8:191. doi: 10.1038/s42004-025-01568-1 (PMC12185727; doi:10.1038/s42004-025-01568-1)
Supplement: Supplementary file 3 — Description of Additional Supplementary Files [file 42004_2025_1568_MOESM3_ESM.pdf]

Description of Additional Supplementary Files

File name: Supplementary Data 1

Description: NMR Spectra and NMR raw files
