# Supplementary material for: Developing a new cleavable crosslinker reagent for in-cell crosslinking
Source: Commun Chem. 2025 Jun 23;8:191. doi: 10.1038/s42004-025-01568-1 (PMC12185727; doi:10.1038/s42004-025-01568-1)
Supplement: Supplementary file 4 — Supplementary Data 1 [file 42004_2025_1568_MOESM4_ESM.zip › Supplementary Data 1.docx]

Dimethyl 5-bromoisophthalate (**S2**)

**^1^H NMR (400 MHz, CDCl_3_)**

(5-Bromo-1,3-phenylene)dimethanol (**S3**)

**^1^H NMR (400 MHz, CDCl_3_)**

(5-Bromo-1,3-phenylene)dimethanol (**S3**)

**^13^C{^1^H} NMR (101 MHz, CDCl_3_)**

1-Bromo-3,5-bis(bromomethyl)benzene (**S4**)

**^1^H NMR (400 MHz, CDCl_3_)**

**^13^C{^1^H} NMR (101 MHz, CDCl_3_)**

Dimethyl 3,3'-(((5-bromo-1,3-phenylene)bis(methylene))bis(sulfanediyl))dipropionate (**S5**)

**^1^H NMR (400 MHz, CDCl_3_)**

**^13^C{^1^H} NMR (101 MHz, CDCl_3_)**

Dimethyl 3,3'-(((5-((trimethylsilyl)ethynyl)-1,3-phenylene)bis(methylene))bis(sulfanediyl))-dipropionate (**S6**)

**^1^H NMR (400 MHz, CDCl_3_)**

**^13^C{^1^H} NMR (101 MHz, CDCl_3_)**

3,3'-(((5-Ethynyl-1,3-phenylene)bis(methylene))bis(sulfanediyl))dipropionic acid (**S7**)

**^1^H NMR (500 MHz, CDCl_3_)**

**^13^C{^1^H} NMR (151 MHz, CDCl_3_)**

Bis(2,5-dioxopyrrolidin-1-yl)3,3'-(((5-ethynyl-1,3-phenylene)bis(methylene)) bis(sulfanediyl))dipropionate (**S8a**)

**^1^H NMR (400 MHz, CDCl_3_)**

**^13^C{^1^H} NMR (151 MHz, CDCl_3_)**

(Bis(1,3-dioxoisoindolin-2-yl) 3,3'-(((5-ethynyl-1,3-phenylene)bis(methylene))bis(sulfanedi-yl))dipropionate (**S8b**)

**^1^H NMR (400 MHz, CDCl_3_)**

**^13^C{^1^H} NMR (101 MHz, CDCl_3_)**

**DiSPASO** (**1**)

**^1^H NMR (600 MHz, CDCl_3_)**

**^13^C{^1^H} NMR (151 MHz, CDCl_3_)**

**DiPPASO** (**2**)

**^1^H NMR (400 MHz, CDCl_3_)**

**^13^C{^1^H} NMR (101 MHz, CDCl_3_)**
